# Death Be Not Proud—Cell Death Control in Plant Fungal Interactions

**DOI:** 10.1371/journal.ppat.1003542

**Published:** 2013-09-12

**Authors:** Martin B. Dickman, Paul de Figueiredo

**Affiliations:** 1 Norman Borlaug Center, Texas A&M University, College Station, Texas, United States of America; 2 Department of Microbial Pathogenesis and Immunology, College of Medicine, Texas A&M Health Science Center, College Station, Texas, United States of America; 3 Department of Veterinary Pathobiology, College of Veterinary Medicine, Texas A&M University, College Station, Texas, United States of America; 4 Department of Plant Pathology and Microbiology, Texas A&M University, College Station, Texas, United States of America; Duke University Medical Center, United States of America

While the concept of programmed cell death (PCD) or its morphological equivalent, apoptosis, was recognized in pockets of research prior to the 1970s, it was not until 1972 that Kerr, Wyllie, and Currie [1] first promulgated the phenomenon. It took nearly 20 years for the original idea to gain acceptance, thanks in large part to seminal studies conducted with *Caenorhabditus elegans*, which provided a solid genetic basis for these observations [2]. These studies also brought forth the idea that cell suicide is central to the life and well-being of multicellular organisms, and is neither uncommon nor normally detrimental to the organism.

Despite the simplicity and finality of the concept of death, the manifold processes by which cells die are not necessarily equivalent. While the role of cell death is firmly established in mammalian disease, it has not been as deeply characterized in other systems. In this Pearl article, we highlight several major ways in which cells die in the context of the high-stakes arms race between fungal pathogens and their plant hosts (Fig. 1).

**Figure 1:**
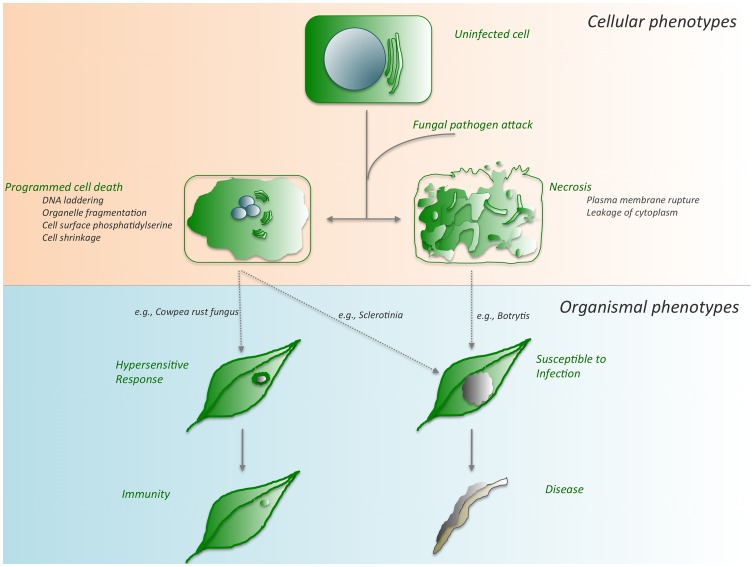
Cell death outcomes following fungal pathogen attack. Depending on the genotypes of both the plant host and the fungal invader, any one of several cell death pathways can be activated. Although these pathways intersect in cell death, they culminate in disparate outcomes, immunity, or disease as detailed in the text. During the recognition of fungal challenge by the plant, host-controlled HR-PCD leads to a restricted cell death phenotype and ultimately immunity. Conversely, pathogen-mediated PCD suppresses this host recognition, leading to unrestricted spread of the pathogen accompanied by PCD, susceptibility, and disease.

## Some Have Called Thee Mighty and Dreadful [3]

The material reality of death can sometimes involve the transition of a mighty living system into dreadful chaos. The process of necrotic cell death exemplifies this notion, and is associated with organelle swelling, a complete disorganization and de-compartmentalization of cellular contents, cell lysis via rupture of the plasma membrane, and leakage of cellular debris into the environment. During necrosis, the dying cell has little to no genetic control of these events. In contrast, apoptotic PCD events unfold in a genetically controlled, highly orchestrated process that includes cell shrinkage, fragmentation and “laddering” of DNA, plasma membrane blebbing, and phosphatidylserine externalization. A hybrid phenomenon, termed “programmed necrosis” (necroptosis) has also been recently described [4]. In this physiologically and pathologically relevant process, first documented in mammalian systems [5], the observed necrotic cell death functions as a back-up system that is activated when the PCD machinery is impaired. In many cases, necroptosis is induced following the initial activation of the extrinsic (receptor-mediated) PCD pathway [6]. Elevations in reactive oxygen species (ROS) are typical of necroptotic cells [7]. As functional parallels exist between plant and animal PCD (see below), it is plausible that necroptosis may constitute a novel hub of plant PCD systems.

Pathways for apoptotic programmed cell death are relatively well characterized in mammals. This is in contrast to plants and phytopathogenic fungi where core regulatory elements of apoptosis pathways have not been found [8]. However, expression of mammalian apoptosis regulators confers phenotypes in plants and fungi that are consistent with their known functions [9]. In addition, numerous studies with baker's yeast (*Saccharomyces cerevisiae*) have shown that this organism exhibits several, but not all, of the hallmarks of apoptosis [10]. Production of ROS, chromatin condensation, DNA fragmentation, and externalization of phosphatidylserine have been shown in yeast, but there is not complete agreement as to whether “true” apoptosis occurs. As is the case in plants, yeast do not possess *bona fide* caspases that possess the precise structural characteristics that define these proteases. However, both yeast and plants harbor distantly related meta-caspases, identified computationally (for a lively discussion of the pros and cons of yeast PCD vs. mammalian PCD, see [10], [11]).

Finally, there have been several comparative studies that examined plant apoptotic-like PCD in the context of biotic and abiotic stress responses, senescence, or other aspects of plant growth and development [12]. Plant and animal cell death regimes clearly displayed differences. For example, plant cells have a rigid cell wall and lack caspases or phagocytic machinery. However, plant and animal PCD share important similarities, including chromatin condensation, DNA laddering, the generation of ROS, and the externalization of phosphatidylserine. Importantly, the underlying conceptual framework for PCD, ranging from development to pathogen attack to abiotic stress, is remarkably conserved for all eukaryotes. Thus, an analysis of cell suicide in plant and animal systems suggests that these processes are observed across kingdoms. For a more detailed discussion of this topic, see [13].

## With Poison, War, and Sickness Dwell [3]

Many necrotrophic plant pathogenic fungi produce phytotoxic metabolites and peptides that play a central role in their pathogenic programs. These “poisons” are used by pathogens to attack their susceptible plant hosts. Phytotoxins can be non-host-specific and thus target a broad range of host plants, or host-specific and thus target a single plant species or even a particular cultivar within a given species [14]. However, while fungal toxins can be simply toxic, increasing evidence suggests that some toxins induce signaling that directs host pathways towards PCD, which exclusively benefits the fungus. For example, the filamentous ascomycete *Cochliobolus victoriae* is a necrotrophic fungal pathogen of *Arabidopsis* and oats and the causative agent of Victoria blight [7], a disease which decimated U.S. oat production in the 1940s. The fungal host–selective toxin victorin is a chlorinated cyclic pentapeptide that elicits several defense responses, PCD, and disease. In *Arabidopsis*, victorin targets a Nod-like NB-LRR-type resistance (R) protein, designated LOV1 [15]. This R protein must be present for fungal susceptibility. The fungus exploits R gene– mediated resistance to incite disease by inducing plant defense responses. Thus, the host R gene, which triggers cell death as a presumable defense, can function as both a resistance and a susceptibility factor. Taken together, the evidence indicates that death-inducing “poisons” can display subtlety in controlling the outcome of fungal pathogen–host interactions.

## Thou Art Slave to Fate [3]

Host-controlled PCD is often required for defense against pathogens. Indeed, the well-studied plant PCD commonly known as the hypersensitive response (HR) is correlated with host defenses that serve to restrict pathogen growth. In the plant host, HR-PCD limits the spread of the pathogen, which is particularly relevant in the case of interactions between biotrophic pathogens and their plant hosts, where host-controlled cell death is correlated with resistance. Conversely, several fungal pathogens appear to promote host cell death, thereby subverting plant cell death pathways for fungal nutrient acquisition. Thus, programmed cell death is a common readout observed during both susceptible and resistant interactions. The eventual victor is decided by which side is in control of cell death, cellular context, and the activities of the combatants. Therefore, the way host cells die provides insight into the eventual fate of the fungal host interaction.

## Poppies and Charms Can Make Us Sleep as Well [3]

The cellular mechanisms that can drive cell death are manifold. Two such “poppies and charms” that can lead to cell death are pyroptosis and autophagy. In animal systems, apoptosis can provide for the “clean” removal of dying cells, with limited induction of inflammatory responses. However, in pyroptosis, cell death is accompanied by the activation of inflammatory signaling. First noted in macrophages [16] and more recently reported in mammalian cells infected with viral or bacterial pathogens [17], pyroptosis typically engages the host inflammasome, a macromolecular complex that senses pathogen-associated molecular patterns (PAMPS) or danger-associated molecular patterns (DAMPS) to drive caspase-1 (and possibly caspase-11) dependent pro-inflammatory cascades [18]. Recently, these ideas have been extended to interactions between fungal pathogens and their susceptible plant hosts [19]. For example, while there are no *bona fide* caspases in plants, plant vacuolar processing enzyme gamma (VPEg) exhibits caspase 1-like activity in plant systems [20], [21]. Plant VPE protease activity is suppressed by caspase-1 specific inhibitors (xVAD-fmk) and is necessary for cell death mediation from a wide range of plant pathogens. The *Fusarium verticilliodes* toxin Fumonisin B1 requires the vacuolar processing enzyme (VPE) for PCD, as VPE mutants prevent mycotoxin-triggered death [22]. Similarly, the model oomycete pathogen, *Hyaloperonospora arabidopsidis,* induces VPE activity during infection of *Arabidopsi*s [23]. This finding is somewhat surprising, as *Hpa* is a biotroph. It was proposed that increased VPE activity may benefit the pathogen by mediating protein turnover and nutrient release. The plant and animal enzymes, while having similar model substrate specificity, clearly display differences. Unlike mammalian caspase 1 which is localized in the cytosol, VPE is localized in the plant vacuole. Therefore, activation of VPE signaling and proteolysis contribute to vacuolar breakdown and apoptosis.

Autophagy can also promote cell death during interactions between fungal pathogens and their susceptible plant hosts. Autophagy was originally understood to be a process whereby starved cells cannibalize their organelles and cytosolic components to promote their survival. However, autophagy as a means for cell survival and homeostasis is now also appreciated as a mechanism to control interactions between fungal pathogens and plants.

The role of plant autophagy in response to fungal pathogens has been investigated in several studies; however, the mechanistic details are incomplete. Autophagy-defective *Arabidopsis* plants were more resistant to the biotrophic fungus *Golovinomyces cichoracearum* in a salicylic acid (SA)-dependent manner [24]. Increased resistance to the virulent biotrophic bacterial pathogen *Pseudomonas syringae* pv. *tomato* was also observed in plants harboring mutations in autophagy proteins [25], [26]. These studies suggest a negative effect of plant autophagy in resistance responses toward biotrophic pathogens and are in contrast to N-mediated resistance to tobacco mosaic virus (TMV), which requires functional autophagic machinery for an effective (HR-PCD) defense response [27]. Thus, in various host pathogen interactions, the consequences of host autophagy-mediated PCD can be diverse.

Although the effect of autophagy on biotrophic interactions is complex and perhaps indirect in its relationship to disease, necrotrophic organisms feed off of dead cells and thus it might be expected that conditions favorable to cell death would promote nectrotrophic growth. Indeed, *Arabidopsis* ATG5, ATG10, and ATG18a mutants developed spreading necrosis and enhanced disease susceptibility upon infection with necrotrophic fungus *Alternaria brassicicola* [25], [26]. This includes elevated ROS, which appeared in noninfected regions as well. Similar runaway cell death symptoms were observed in the autophagy mutants after application of fumonisin B1. In contrast, no difference in growth characteristics was observed for the obligate biotroph *H. arabidopsidis* [25]. Thus, runaway (unrestricted) cell death in the mutants indicates that autophagy plays an anti-death role, perhaps by limiting ROS effects and restricting pathogen spread. Indeed, elevated spontaneous ROS accumulation is readily observed in autophagy mutants [28]. Further support for susceptibility to nectrotrophic lifestyles was shown by enhanced rates of infection by the necrotrophic pathogen *Botrytis cinerea* in AtATG18a mutants [29]. Not unexpectedly, the necrotrophic pathogen *B. cinerea* has been reported to benefit from PCD [30]. Thus, cell death induced by autophagy might be expected to result in enhanced fungal growth. It is reasonable that a necrotroph would benefit from a local environment of dead tissue. However, this is clearly not the case, as *B. cinerea* triggers autophagous cell death, resulting in a restricted growth phenotype (Dickman, unpublished observations). We interpret these observations to indicate that the process by which host cells die (i.e., the means by which death occurs) is key for determining the ultimate outcome of interactions between host and pathogen [31].

## And Death Shall Be No More; Death, Thou Shalt Die [3]

The struggle between plant hosts and their fungal pathogens to control the mechanisms by which host cells die can have profound effects on the ultimate outcome of the interaction. With this idea in mind, it is notable that, to date, very few examples of fungal anti-death factors have been described. It has been shown that *B. cinerea* undergoes massive PCD following penetration of the host plant and establishment of a primary necrotic lesion [32]. The host defense molecule camalexin was shown to be capable of triggering PCD in *B. cinerea*. In response, the fungus suppresses this host-induced PCD to establish infection. Despite these intriguing observations, the anti-death factors that mediate the suppression of host PCD in this system have yet to be identified. The identification of factors that suppress host cell death in necrotrophic fungal pathosystems (e.g., see [33]) constitutes an important line of future investigation.
